# Prevalence and Associated Risk Factors of Primary Open Angle Glaucoma Among Patients With Type 2 Diabetes Mellitus: A Cross-Sectional Study From North India

**DOI:** 10.7759/cureus.28908

**Published:** 2022-09-07

**Authors:** Wahegurupal Singh, Nitin Singh Salaria, Mohan Lal Pandey, Vipan Bhandari, Simranpreet Singh, Prashant Bhardwaj

**Affiliations:** 1 Ophthalmology, Maharishi Markandeshwar Institute of Medical Science and Research, Ambala, IND; 2 Forensic Department, Maharishi Markandeshwar Institute of Medical Science and Research, Ambala, IND; 3 Pharmacology, Maharishi Markandeshwar Institute of Medical Science and Research, Ambala, IND; 4 Ear Nose Throat and Head Neck Surgery, SSB Heart and Multispeciality Hospital, Faridabad, IND

**Keywords:** type 2 diabetes mellitus, visual field, optic disc, anterior chamber depth, primary open angle glaucoma

## Abstract

Background

Glaucoma is a category of disorders that cause visual loss due to damage to the optic nerve. It is the primary cause of irreversible blindness around the globe. In diabetic individuals, intraocular pressure (IOP) was found to be higher than in non-diabetics, and this impact was linked to a rise in fasting blood glucose levels. In light of the foregoing findings, the current investigation was carried out with the goal of determining the prevalence and associated factors for primary open-angle glaucoma in people with diabetes in a tertiary care teaching hospital.

Methods

This cross-sectional study was carried out in a tertiary care teaching hospital. For a twelve-month period (2021-2022), adult patients (with type 2 diabetes and an age of 18 years or more) attending the ophthalmology out patient department (OPD) were included. Demographic information, socioeconomic data, and medical, familial, and ocular history were all gathered. All individuals with diabetes had an initial ophthalmological evaluation [including best corrected visual acuity (BCVA), slit-lamp biomicroscopy, assessment of peripheral anterior chamber depth (ACD), fundus examination, stereoscopic optic disc evaluation, and IOP measurement] by a senior ophthalmologist. Pearson's Chi-square test was used to compare variables on a nominal scale, whereas the t-test for independent samples was used to examine variables on a continuous scale.

Results

Our study enrolled a total of 1262 diabetic patients out of which 62.0% of subjects were male. Most of the enrolled subjects belonged to the 61-70 years of age group (35.8%). The eye examination of enrolled subjects showed that out of 1262 diabetic patients, 197 subjects (15.6%) were having primary open-angle glaucoma (POAG). The vision (in the better eye) was analyzed among three groups [no glaucoma or sight-threatening diabetic retinopathy (STDR); only glaucoma; and glaucoma plus STDR] and it was observed that no perception of light (PL) was noticed in 0.2% and 1.2% of subjects in the “no glaucoma or STDR” and “only glaucoma” groups respectively. In our study, the variables significantly associated with the increased prevalence of glaucoma among diabetic subjects included illiteracy, family history of type 2 diabetes mellitus (DM), being hypertensive, non-intake of anti-hypertensive medication, and >10 years duration of DM (p<0.05).

Conclusion

From our study, we came to the conclusion that there is clear-cut evidence of an increased incidence of POAG in diabetic patients, which was 15.6%. The study also showed a significant association between age, education, diet, hypertension, family history of diabetes and duration of diabetes, and; POAG. However, no significant association was found between gender and POAG.

## Introduction

Glaucoma is a category of disorders that cause visual loss due to damage to the optic nerve. It is the primary cause of irreversible blindness around the globe. The most frequent types of glaucoma are open-angle and angle-closure glaucoma. Both can be separated into primary and secondary causes, as well as acute, subacute, and chronic stages of progression. The patient's quality of life, physical capabilities, and mental well-being are all affected by the group of diseases [1,2].

Primary open-angle glaucoma is a persistent, condition that affects both eyes. In the absence of recognized causes, the diseases are defined by structural abnormalities of the optic disc or retinal nerve fiber layer, as well as an open anterior chamber. Adults are affected by the POAG, and may or may not have vision loss. Higher intraocular pressure, advanced age, family history, and African heritage are all risk factors connected with the illness. Traditionally, increased IOP was assumed to be the primary cause of ocular neuropathy. In up to 40% of instances of open-angle glaucoma, the intraocular pressure is normal or low [3].

The importance of early detection cannot be overstated, as it directly impacts the visual or functional outcome. Treatment approaches can assist reduce progression in both early and late stages of the disease; nonetheless, the visual impairment is permanent [4]. Iatrogenic glaucoma is the most common cause of secondary open-angle glaucoma. Eye medications including corticosteroids, ocular surgery, and laser are some of the reasons.

Patients with diabetes have a higher risk of developing glaucoma than those who do not have diabetes. Patients who had been diagnosed for longer periods were at a higher risk. In diabetes individuals, IOP is found to be higher than in non-diabetics, and this impact is linked to a rise in fasting blood glucose [5,6]. In light of the foregoing findings, the current investigation was carried out to determine the prevalence and associated factors for primary open-angle glaucoma in people with diabetes in a tertiary care teaching hospital.

## Materials and methods

This cross-sectional research was carried out in a tertiary care teaching hospital. The approval of the Institutional Ethics Committee was obtained (IEC number: MMIMSR/2020/418). For a twelve-month period (2021-2022), adult patients (with type 2 diabetes and an age of 18 years or more) attending the ophthalmology out patient department (OPD) were included. All participants signed a written informed consent form. To detect the cases of primary glaucoma, all diabetic patients who were referred for cataract surgery or for the diagnosis and management of other ocular diseases such as glaucoma, diabetic retinopathy, age-related macular degeneration, pterygium, and secondary glaucoma underwent a detailed ophthalmological examination. Patients having congenital or juvenile glaucoma, as well as secondary glaucoma (lens-induced, pseudo-exfoliation, neovascular, and uveitis), were not included in the study.

Demographic information, socioeconomic data, and medical, familial, and ocular history were all gathered. All individuals with diabetes had an initial ophthalmological evaluation (senior ophthalmologist) that included recording best corrected visual acuity (BCVA) using Snellen's chart at 6 m, slit-lamp biomicroscopy (Topcon, Oakland, NJ, USA), assessment of peripheral anterior chamber depth (ACD) using Van-Herick technique, fundus examination using indirect ophthalmoscopy, stereoscopic optic disc evaluation using +78D lens (x16 magnification), and IOP measurement using Goldman’s applanation tonometer.

Probable glaucoma patients were directed to a glaucoma specialist for additional glaucoma workup, including gonioscopy, disc evaluation, and visual field testing. A four-mirror Sussman gonioscope (Ocular Instruments, Inc., Bellevue, WA) was used to perform gonioscopy, and the angle was graded using modified Shaffer's classification. The vertical cup disc ratio (VCDR) was estimated, and glaucomatous optic neuropathy morphological abnormalities (including glaucomatous cupping) were identified. If the IOP was less than 21 mmHg or the VCDR was less than 0.7 in either eye, the visual field test was performed [6]. Even if the VCDR difference between the two eyes was >0.2 with the existence of a glaucomatous disc, a visual field test was performed to rule out primary glaucoma. When feasible, a visual field test was attempted using a Humphrey Field Analyzer (SITA 24-2; Humphrey Field Analyzer II; Carl Zeiss Meditec, Inc., Oberkochen, Germany).

The patients were divided into three categories according to the International Society Geographical and Epidemiological Ophthalmology (ISGEO) classification system. Category 1 required structural and functional evidence i.e., 97.5th percentile of the VCDR (≥0.7) or VCDR asymmetry (≥0.1) in our normal population and visual field loss typical of glaucoma. Category 2 required advanced structural damage i.e., 99.5th percentile VCDR (≥0.75) or VCDR asymmetry (≥0.2) in the absence of visual field evidence i.e. when a useful visual field result was not possible or available. Category 3 applied when the optic disc was not seen and visual field testing was not possible and used: a) blindness (VA <3/60) with the 99.5th percentile IOP (≥28 mmHg), or b) diagnosed with/being treated for glaucoma. An additional level of evidence (level2b) was added where the optic disc was visualized but the VCDR was <99.5th percentile and visual fields were not available or if visual fields were interpreted as “unlikely glaucoma” but there was other compelling evidence such as RAPD, high IOP and/or corneal edema. 

If the glaucoma hemifield test graded “outside normal limits” and a cluster of three contiguous points at the 5% level on the pattern deviation plot, using the threshold test strategy with the 24-2 test pattern of the Zeiss-Humphrey field analyzer II, the glaucomatous visual field defect was considered to be present [3]. The presence of two or more of the following findings was used to characterize glaucoma: (1) Any focal notch in the neuro-retinal rim; (2) thinning and pallor of the neuro-retinal rim; (4) hemorrhage on the optic disc; (5) peripapillary atrophy; (6) visual field defects correlating with optic disc changes; (7) mean deviation >6 db and pattern standard deviation >3 db on perimetry; (8) IOP >21mm Hg. In our study, we used the ETDRS classifications for grading the severity of retinopathy. Patients were classified as having STDR or non-sight-threatening diabetic retinopathy (NSTDR) [6]. So, we categorized patients into three 3 groups i.e. only glaucoma; glaucoma plus sight-threatening diabetic retinopathy (STDR), and no glaucoma or no STDR for analysis.

A participant was classified as hypertensive if their systolic blood pressure was greater than 140 mmHg or their diastolic blood pressure was greater than 90 mmHg, or if they had a self-reported history of hypertension.

Statistical analysis

The dataset of primary glaucoma patients at the hospital was evaluated, and descriptive statistics such as mean, standard deviation, and percentage for the study variables were calculated using the measuring scale. These tests were carried out based on whether the patients were glaucomatous or non-glaucomatous. Pearson's Chi-square test was used to compare variables on a nominal scale, whereas the t-test for independent samples examined variables on a continuous scale. The statistical significance was determined at a 5% level, and the full analysis was carried out with SPSS (IBM Corp. Released 2011. IBM SPSS Statistics for Windows, Version 20.0. Armonk, NY: IBM Corp).

## Results

**Figure 1:**
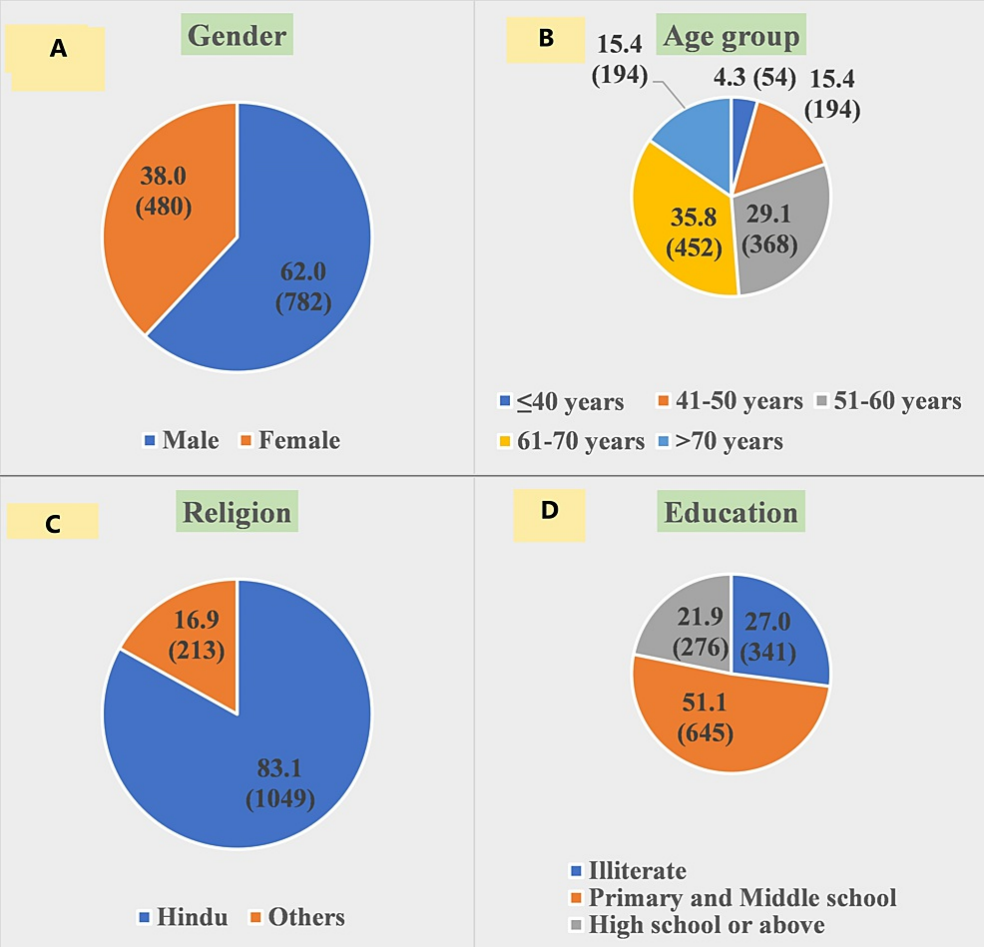
Sociodemographic profile of enrolled subjects (N=1262). (A) Gender distribution of subjects. (B) Age distribution of subjects. (C) Religion wise distribution of subjects. (D) Education status of subjects.

Our study enrolled a total 1262 diabetic patients out of which 62.0% of subjects were male. Most of enrolled subjects belonged to 61-70 years of age group (35.8%). More than one fourth of subjects were illiterate (27.0%) and less than one fourth were educated up to high school or above (21.9%) (Figure 1).

**Figure 2:**
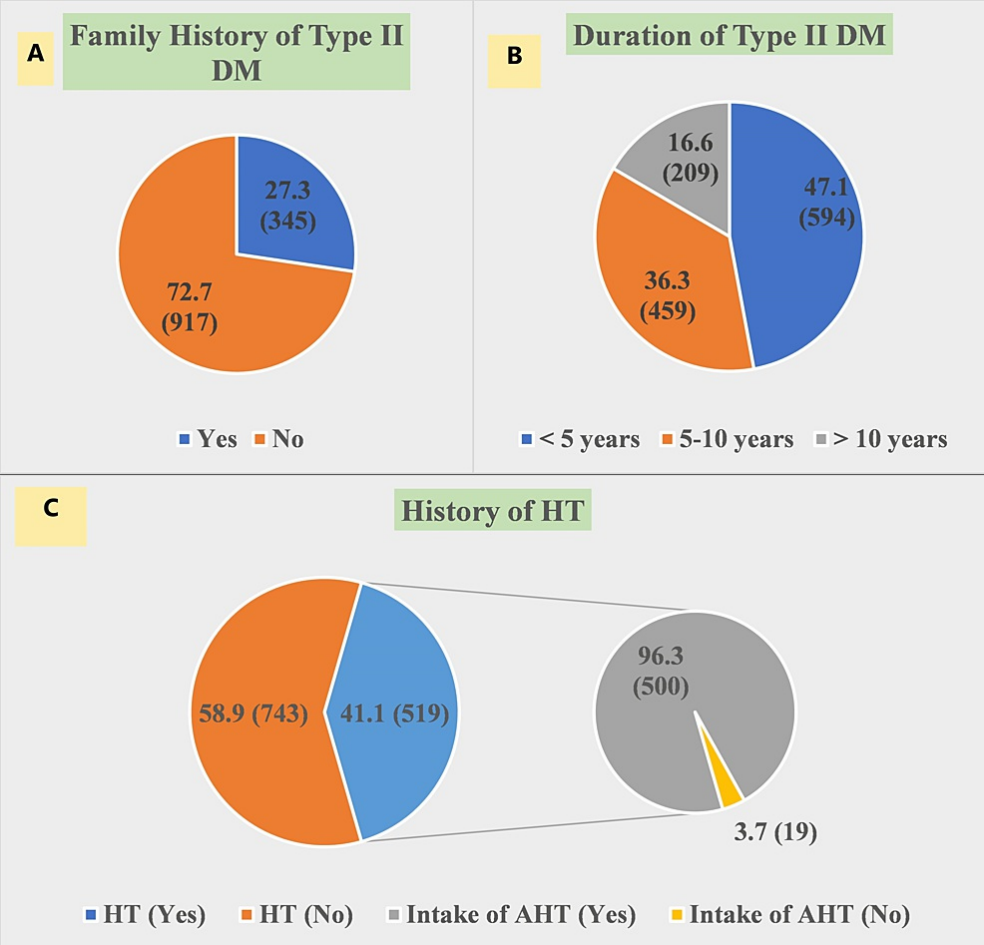
Clinical history of subjects (N=1262). (A) Family history of Type II diabetes mellitus (DM) among subjects. (B) Duration of diabetes mellitus (DM) among subjects. (C) History of hypertension (HT) and its treatment among subjects DM= Diabetes mellitus, HT= Hypertension, AHT= Antihypertensive

Figure 2. shows that 27.3% of subjects gave family history of type 2 DM. The duration DM among enrolled subjects was <5 years for 47.1% of subjects, 5-10 years for 36.3% of subjects, and > 10 years for 16.6% subjects. Also, 41.4% of subjects gave history of hypertension (HT) and among them nearly all subjects (96.3%) were having intake of antihypertensive medication.

**Figure 3:**
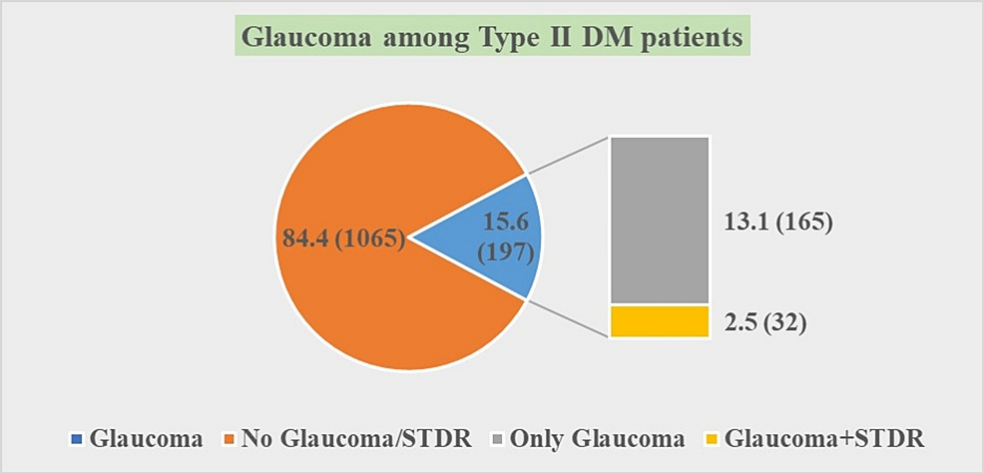
Prevalence of glaucoma and sight-threatening diabetic retinopathy (STDR) among subjects (N=1262). DM: diabetes mellitus, STDR: sight-threatening diabetic retinopathy

The eye examination of enrolled subjects showed that out of 1262 diabetic patients (Figure 3), 197 subjects (15.6%) were having primary open angle glaucoma (POAG) [only glaucoma (13.1%) and glaucoma plus sight-threatening diabetic retinopathy (STDR) (2.5%)].

**Figure 4:**
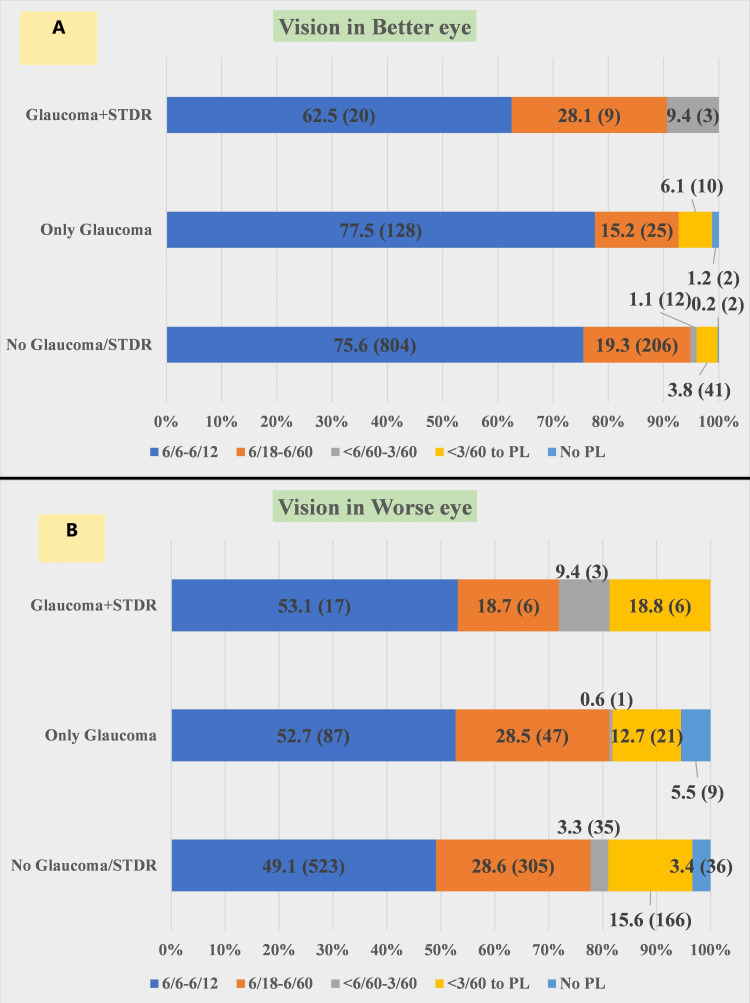
Distribution of vision among subjects (N=1262). (A) The vision (in the better eye) among three groups (no glaucoma or STDR; only glaucoma; and glaucoma plus STDR). (B) The vision (in the worse eye) among three groups (no glaucoma or STDR; only glaucoma; and glaucoma plus STDR). STDR: sight-threatening diabetic retinopathy, PL: Perception of light

The vision (in the better eye) was analyzed among three groups (no glaucoma or STDR; only glaucoma; and glaucoma plus STDR) and it was observed that no perception of light (PL) was noticed in 0.2% and 1.2% of subjects in the “no glaucoma or STDR” and “only glaucoma” groups respectively. The vision of <3/60 to PL was observed among 3.8% and 6.1% of subjects in the “no glaucoma or STDR” and “only glaucoma” groups respectively. The vision (in the worst eye) was analyzed among three groups (no glaucoma or STDR; only glaucoma; and glaucoma plus STDR) and it was observed that no perception of light (PL) was noticed in 3.4% and 5.5% of subjects in the “no glaucoma or STDR” and “only glaucoma” groups respectively. The vision of <3/60 to PL was observed among 15.5%, 12.7%, and 18.8% of subjects in the “no glaucoma or STDR”, “only glaucoma”, and “glaucoma plus STDR” groups respectively (Figure 4).

In our study (Table 1), the variables significantly associated with the increased prevalence of glaucoma among diabetic subjects included illiteracy, family history of type 2 DM, being hypertensive, non-intake of hypertensive medication, and >10 years duration of DM (p<0.05). The prevalence of glaucoma decreased with the increase in the education level (Illiterate: 21.7% vs Primary and Middle school: 16% vs High school or above: 13.4%). The prevalence of glaucoma was higher among subjects with a family history of type 2 DM (19.1%) as compared to those without a family history of type 2 DM (14.3%). Also, the prevalence of glaucoma increased with the increase in the duration of DM (<5 years: 11.4% vs 5-10 years: 19.0% vs >10 years: 20.1%).

**Table 1:** Association of sociodemographic and clinical profile with glaucoma among subjects (N=1262).

Variables	Glaucoma [Number (%)/Mean±SD]		P Value
	Absent (n=1065)	Present (n=197)	
Gender			
Male	654 (83.6)	128 (16.4)	0.343
Female	411 (85.6)	69 (14.4)	
Age group	55.39±9.72	59.23±8.89	<0.0001
Religion			
Hindu	888 (84.7)	161 (15.3)	0.569
Others	177 (83.1)	36 (16.9)	
Education			
Illiterate	267 (78.3)	74 (21.7)	0.016
Primary and Middle school	452 (84)	86 (16)	
High school or above	239 (86.6)	37 (13.4)	
Diet			
Vegetarian	477 (89)	59 (11)	0.0001
Mixed	588 (81)	138 (19)	
Family history of type 2 DM			
Yes	279 (80.9)	66 (19.1)	0.034
No	786 (85.7)	131 (14.3)	
Hypertensive			
Yes	421 (81.1)	98 (18.9)	0.007
No	644 (86.7)	99 (13.3)	
Intake of Antihypertensives			
Yes	410 (82.0)	90 (18.0)	0.008
No	11 (57.9)	8 (42.1)	
Duration of DM			
< 5 years	526 (88.6)	68 (11.4)	0.0005
5-10 years	372 (81.0)	87 (19.0)	
> 10 years	167 (79.9)	42 (20.1)	

## Discussion

According to the World Health Organization, India has 8.9 million blind persons, with glaucoma accounting for 12.8 percent [7]. Despite its public health importance, data on glaucoma prevalence and potential risk factors in India are scarce [8]. It's critical to get a diagnosis as soon as possible so that treatment can begin to slow the disease's progression. In cases of POAG, a number of risk variables have been found to be significant [9]. However, statistics on the relationship between diabetes and POAG are the need of the hour.

In this study, the mean age of patients with POAG and those without POAG was found to be 59.23±8.89 and 55.39±9.72 years, respectively, which was similar to earlier population-based studies conducted in India. According to the Mitchell et al. study, where the mean age of the study group was 66.2±98 years and that of POAG was 75.9±8.6 years [10], age appears to be an independent risk factor for the development of POAG. In our study, the average age of POAG patients was higher than that of non-glaucomatous diabetic individuals.

The possibility of a link between diabetes and POAG has sparked debate. Diabetes mellitus is more common in patients with POAG than in the non-glaucomatous population, according to research by Becker et al. [10]. Similarly, diabetics have a higher prevalence of POAG than non-diabetics [11]. In our research, we discovered that 15.6 percent of diabetics have POAG. In our study, males (16.4 percent) had higher levels of POAG than females (14.4 percent); which was coherent with a study conducted by Leske et al. [12].

There appears to be a causal link between DM and POAG. Several suggestions have been offered about the biochemical links between DM and POAG. For instance, there is mounting evidence that long-term hyperglycemia, together with lipid abnormalities, may enhance the risk of neuronal injury from stress. Laboratory data, in particular, has provided strong support for such a link [13,14,15].

Furthermore, investigations have shown that diabetic eyes have a diminished ability to self-regulate blood flow and have lower retinal blood flow [16]. As a result, they exhibit relative hypoxia and hypoxia-inducible factor-1 (HIF-1) overexpression [17,18]. In human glaucomatous eyes, levels of HIF-1 rose in ganglion cells, the retina, and the optic nerve head in response to increasing IOP [19]. Another crucial link between DM and POAG could be these.

Lastly, the apparent link between DM and POAG could be explained by the remodeling of the optic nerve head's connective tissue. The remodeling may diminish compliance at the trabecular meshwork and lamina cribrosa, resulting in higher IOP and more mechanical stress on the optic nerve head [20,21]. Diabetes has been shown to increase connective tissue remodeling and enhance these biomechanical alterations in studies [22]. More crucially, the study by Hennis et al. discovered that diabetes was a risk factor for higher IOP in the long run [23]. Sommer et al. observed little or no link between glaucoma and diabetes, whether insulin-dependent (DM type 1) or non-insulin-dependent (DM type 2) [24].

Glaucoma with sight-threatening diabetic retinopathy (STDR) was found in 2.5 percent of the participants in our study. Studies have found a correlation between the severity of diabetic retinopathy and the later occurrence of POAG [15,16]. Many studies have found that diabetics have higher mean IOP and POAG than the general population [20,21]. Glaucomatous patients have also been shown to have greater aberrant glucose levels than the general population [17,18]. In our study, the prevalence of glaucoma rose as the duration of diabetes increased (5 years: 11.4 percent vs 5-10 years: 19.0 percent vs > 10 years: 20.1 percent).

Limitations

The limitation of the study was that it was conducted in diabetic patients only, and there was no control group (non-diabetics), to compare the prevalence of POAG in diabetic and non-diabetic patients. Along with this, the study being single-centric might hinder the generalizability of the results to the non-diabetic population. 

## Conclusions

Primary open-angle glaucoma is mostly asymptomatic until significant visual field loss has occurred. Patients usually present with significant visual field loss in one eye and advanced disease in the other eye. It is associated with irreversible blindness. Thus, the public health importance of detecting undiagnosed and treatable glaucoma, as blindness has economic and societal consequences for the rest of an individual’s life. Several studies have shown an association between POAG and diabetes. From our study, we came to the conclusion that there is clear-cut evidence of an increased incidence of POAG in diabetic patients, which was 15.6%. The study also showed a significant association between age, education, diet, hypertension, family history of DM type 2, and duration of DM type 2 and; POAG. However, no significant association was found between gender and POAG.
